# Au/Pd core-shell nanoparticles with varied hollow Au cores for enhanced formic acid oxidation

**DOI:** 10.1186/1556-276X-8-113

**Published:** 2013-03-01

**Authors:** Chiajen Hsu, Chienwen Huang, Yaowu Hao, Fuqiang Liu

**Affiliations:** 1 Department of Materials Science and Engineering, University of Texas at Arlington, 501 West First Street, Room 231, Engineering Laboratory Building, Arlington, TX 76019, USA

**Keywords:** Electrochemistry, Activity, AuPd, Formic acid oxidation, Core-shell, Fuel cell

## Abstract

A facile method has been developed to synthesize Au/Pd core-shell nanoparticles via galvanic replacement of Cu by Pd on hollow Au nanospheres. The unique nanoparticles were characterized by X-ray diffraction, X-ray photoelectron spectroscopy, transmission electron microscopy, ultraviolet–visible spectroscopy, and electrochemical measurements. When the concentration of the Au solution was decreased, grain size of the polycrystalline hollow Au nanospheres was reduced, and the structures became highly porous. After the Pd shell formed on these Au nanospheres, the morphology and structure of the Au/Pd nanoparticles varied and hence significantly affected the catalytic properties. The Au/Pd nanoparticles synthesized with reduced Au concentrations showed higher formic acid oxidation activity (0.93 mA cm^-2^ at 0.3 V) than the commercial Pd black (0.85 mA cm^-2^ at 0.3 V), suggesting a promising candidate as fuel cell catalysts. In addition, the Au/Pd nanoparticles displayed lower CO-stripping potential, improved stability, and higher durability compared to the Pd black due to their unique core-shell structures tuned by Au core morphologies.

## Background

Polymer electrolyte membrane fuel cells have been considered as potential energy sources to replace batteries for mobile devices. Recently, Pd and Pd-based materials have attracted a lot of attention due to their superior catalytic activities on formic acid oxidation (FAO) [1-11] in direct formic acid fuel cells (DFAFCs) [12-19]. These Pd-based catalysts are synthesized in different structures such as bimetallic alloys [20-23], nanodendrites [23], core-shell [24,25], and nanoneedle [26] through the geometric and electronic effects, the most well-known factors [27] that affect the catalytic reactions and usually work jointly.

Among the developed structures, the core-shell structures of Pd-based materials [28-31] not only demonstrate high catalytic activity, stability, and durability but also provide a suitable platform to understand the interaction between the core and Pd shell. Particularly, Au/Pd core-shell nanoparticles (NPs) are reported to show excellent electrochemical properties in FAO [28,29,31] and oxygen reduction reaction [32]. The catalytic ability dictated by both the geometric and electronic effects in the core-shell structures can be easily tuned by controlling the composition [33], structure, or even particle size of the Au core and Pd layers. Despite extensive development, however, reports on the impact of porous and hollow Au cores in the Au/Pd core-shell structure are rare.

We have developed a unique electrodeposition method to synthesize the Au/Pd core-shell NPs by coating Pd on the surface of hollow Au nanospheres [24]. In this paper, we aim to investigate the impact of the Au support, whose structure has been tuned systemically by adjusting the concentration of the Au solution, on the catalytic ability of the Pd layer toward FAO.

## Methods

The hollow Au/Pd core-shell NPs were fabricated from hollow Au spheres via an electrodeposition method. The electrochemically evolved hydrogen nanobubbles reduced Au^+^ ions at the boundary into metallic hollow Au clusters. The process has been reported in our previous studies [34,35], and the size of the hollow Au nanospheres is between 120 and 180 nm with individual grain size ranging from 2 to 8 nm. To adjust the concentration of the Au solution, a buffer solution, containing sodium sulfite (10%), ethylenediamine (5%), and distilled water (85%), was chosen to dilute the Au solution and keep the Au complex (Na_3_Au(SO_3_)_2_) stable. In this study, we prepared three different hollow Au nanospheres denoted as Au100, Au50, and Au25, in which the number stands for the percentage of the Au concentration relative to the received Au solution (7.775 g L^-1^ from Technic, Woonsocket, RI, USA).

To form the Pd shell onto the hollow Au nanosphere substrates, the Au layers were first coated with Cu in a Cu electroless electrolyte, which consisted of 0.4 M CuSO_4_, 0.17 M ethylenediaminetetraacetic acid disodium salt dehydrate as complexant, and formaldehyde as reducing agent at pH = 10.3 for 10 min. Then an aqueous solution of 2.53 mM PdCl_2_ was used to replace of the Cu layer through a galvanic reaction for 30 min: Cu(s) + Pd^2+^(aq) → Pd(s) + Cu^2+^(aq).

The structures of the NPs were determined using powder X-ray diffraction (XRD) with Cu-Kα source (Siemens D500, Munich, Germany). The scan range was from 30° to 90° at a rate of 1° min^-1^. X-ray photoelectron spectroscopy (XPS) (Perkin-Elmer Phi 560 XPS/Auger System, Waltham, MA, USA) was adopted with Al as the source to explore the electronic structures on the NP surface. High-resolution transmission electronic microscopy (HRTEM) with 300 kV of accelerated voltage (Hitachi H-9500 HRTEM, Tokyo, Japan) was employed to study the NP size and structure. Ultraviolet–visible (UV–vis) spectra were obtained with a Perkin-Elmer Lambda 19 UV/VIS/NIR spectrometer at room temperature. The compositions (weight fractions of Pd to the total mass) of the Au/Pd catalyst determined by inductively coupled plasma mass spectrometry (Perkin-Elmer ELAN DRC II) are as follows: Au25Pd 36.4 wt.%, Au50Pd 32.5 wt.%, and Au100Pd 29.5 wt.%, respectively.

Electrochemical measurements were conducted with a Princeton Applied Research 2273 potentiostat (Oak Ridge, TN, USA) in three-electrode configuration using a rotating disk electrode (Pine Instrument Company, Grove City, PA, USA) of glassy carbon (area, 0.19635 cm^2^). Pt mesh and normal hydrogen electrode (NHE) were used as the counter electrode and reference electrode, respectively. Preparation of catalyst inks and rotating disk electrodes followed the procedure described in our previous work [18,24]. Typically, 15 mg of catalysts was mixed with 12 ml of distilled water and 4.4 ml of 5 wt.% Nafion (Ion Power Inc., Newcastle, DE, USA) and then dried under an IR lamp. During the CO-stripping measurement, CO was first adsorbed to the catalysts at 0.2 V for 900 s in CO-saturated 0.1 M HClO_4_ solution, and then cyclic voltammetry (CV) was conducted from 0.075 to 1.2 V at a rate of 10 mV s^-1^ after bubbling Ar for an hour. The FAO test was conducted in a mixed electrolyte (0.1 M HClO_4_ and 0.1 M HCOOH) from -0.03 to 1.4 V (vs. NHE) at 1,000 rpm. The catalytic durability was tested by an accelerated stress test (AST) protocol with square-wave potential cycles between 0.6 V (5 s) and 0.95 V (5 s).

## Results and discussion

**Figure 1:**
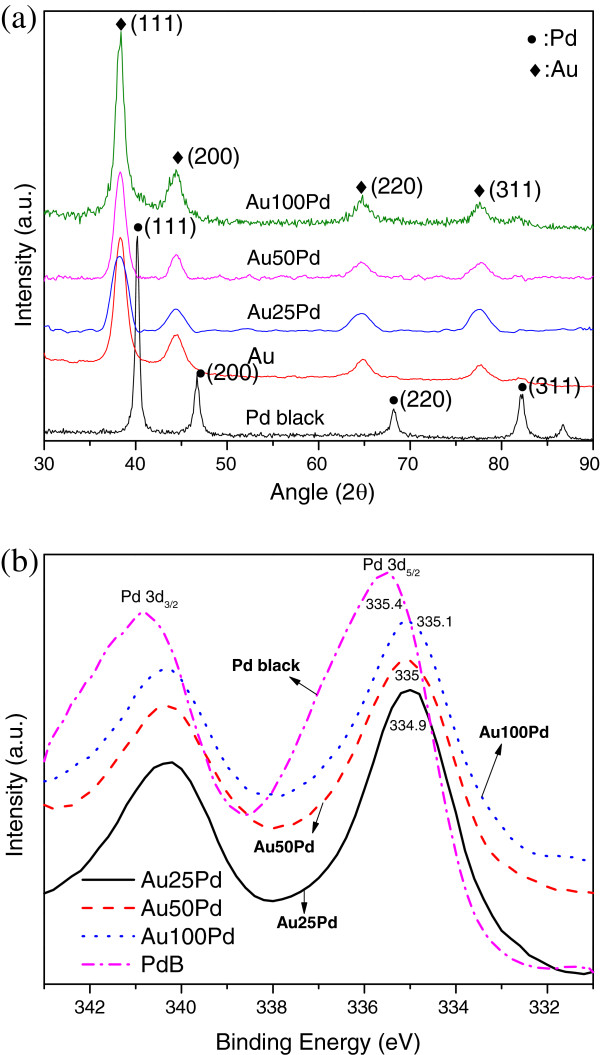
**Structures of the nanoparticles. **(**a**) X-ray diffraction patterns of the Au/Pd, Au, and Pd black nanoparticles and (**b**) Pd 3*d *XPS spectra of the Au/Pd catalysts and Pd black.

**Figure 2:**
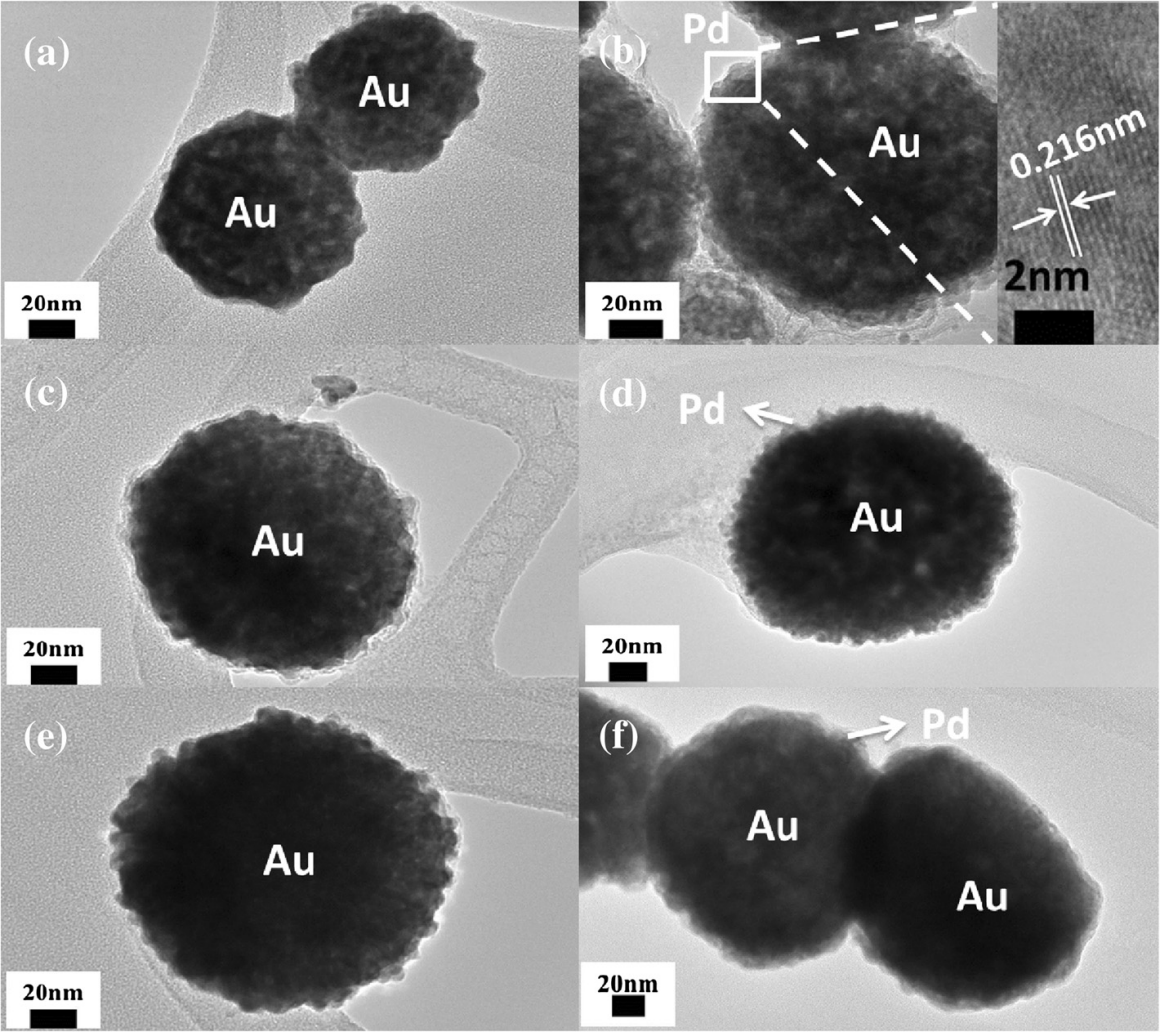
**TEM images. **(**a**) Au25, (**b**) Au25Pd with the inset showing the Pd nanocrystallites from the Pd shell, (**c**) Au50, (**d**) Au50Pd, (**e**) Au100, and (**f**) Au100Pd. The obvious dark/white contrast identified in the images of the Au nanospheres indicates that they are porous.

**Figure 3:**
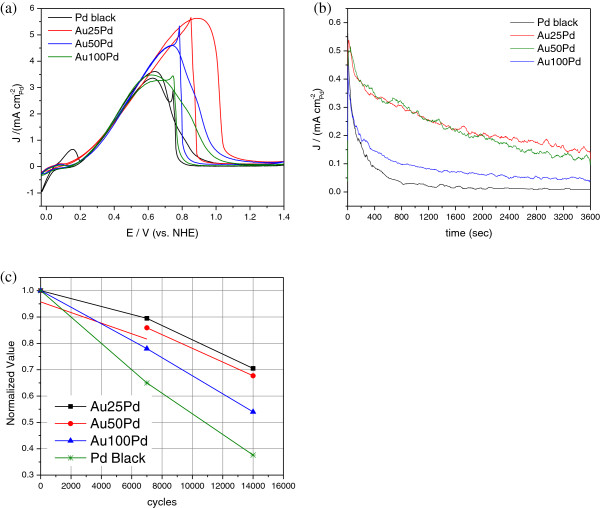
**FAO test results.** (**a**) FAO CV of the Au/Pd and Pd black catalysts in 0.1 M HClO_4 _and 0.1 M HCOOH solution from -0.03 to 1.4 V and rotated at 1,000 rpm. The area-specific current densities of the Au25Pd, Au50Pd, Au100Pd, and Pd black are normalized to the ECSA. (**b**) Chronoamperometry curves of the Au/Pd and Pd black nanoparticles in 0.1 M HClO_4 _and 0.1 M HCOOH solution at 0.3 V up to 3,600 s. (**c**) Relative ECSA losses for the Au/Pd and Pd black nanoparticles in 0.1 M HClO_4 _solution during potential-cycling tests at the potential step between 0.95 V and 5 s and 0.6 V and 5 s, recorded at 7,000 cycles (19.4 h) and 14,000 cycles (38.89 h).

Figure 1a shows the XRD reflection peaks of the Pd black (Alfa Aesar, Ward Hill, MA, USA) at 40.20°, 46.73°, 68.21°, and 82.17°, respectively, corresponding to Pd (111), (200), (220), and (311) planes in the fcc structure (JCPDS no. 87–0639; 40.21°, 46.78°, 68.3°, and 82.34°). For the Au/Pd NPs, the peaks at 38.28°, 44.42°, 64.76°, and 77.81° correspond respectively to Au (111), (200), (220), and (311) planes (JCPDS no. 65–2870; 38.19°, 44.38°, 64.57°, and 77.56°). The very weak Pd signal in the XRD pattern is attributed to the highly dispersed Pd nanocrystallites embedded at the Au surface (shown in the TEM images in Figure 2). There is no identifiable Cu signal in the spectra, suggesting that Cu has been completely replaced by the Pd shell, which confirms our previous study [24]. Besides, Figure 1b displays the Pd 3*d* core-level XPS spectra of the Au/Pd NPs and Pd black. The Au25Pd NPs showing lower Pd 3*d*_5/2_ binding energy (334.9 eV) might demonstrate higher activity than other Au/Pd NPs (Au50Pd, 335 eV; Au100Pd, 335.1 eV) and the Pd black (335.4 eV), which will be confirmed in Figure 3.

The microstructures of the hollow Au and Au/Pd NPs were studied by a high-resolution TEM, and Figure 2 shows the images of both the Au and Au/Pd NPs synthesized using different concentrations of Au solutions. Figure 2a,b shows the TEM images of the Au25 and the corresponding Au/Pd NPs (i.e., Au25Pd), respectively. The images clearly display porous Au structures (identified by contrast of the TEM images) with 100-nm diameter and Pd shells with a thickness of 5 to 10 nm. The inset in Figure 2b shows the HRTEM image of the Pd outer shell which indicates crystalline nature with a *d* spacing of 0.216 nm (refer to JCPDS no. 87–0639; *d* = 0.224 nm). Figure 2c,d, showing the TEM images of the Au50 and Au50Pd, indicates that their sizes are around 115 and 130 nm in diameter, respectively. In addition, Figure 2e,f shows the Au100 with 126-nm diameter and Au100Pd with 145-nm diameter. The comparison of these TEM images indicates that Au25 has the smallest particle size and the most porous structure than others. With increasing Au concentration, the porosity of the Au nanospheres decreases, but the size continuously grows almost linearly due to the increased Au solution concentrations.

**Figure 4:**
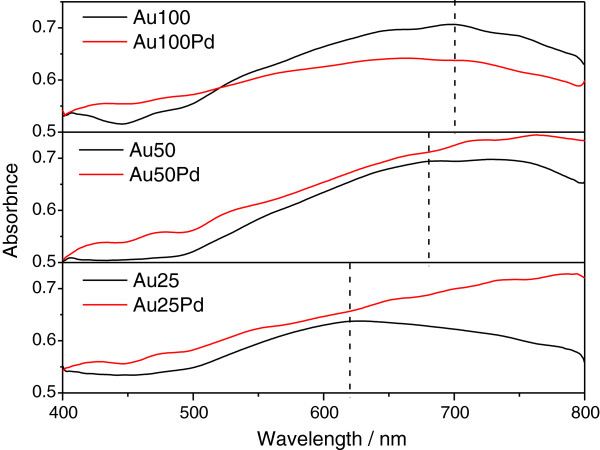
UV–vis absorption spectra of different Au and Au/Pd nanoparticles.

UV–vis studies were performed to probe the surface coverage of Pd on the NPs. Figure 4 shows the absorption spectra of the Au and Au/Pd NPs and indicates that the absorption peak increases from 616 nm (Au25) to 698 nm (Au50 and Au100) due to the surface plasmon resonance effect of Au. The Au/Pd NPs also reveal absorption peaks around 700 nm with the Au100Pd being more pronounced, indicating that the Pd shell does not fully cover the Au core surface. This observation is in agreement with the studies carried out by of Shim et al. [32] because Pd does not show any absorption peak within the wavelength studied.

**Figure 5:**
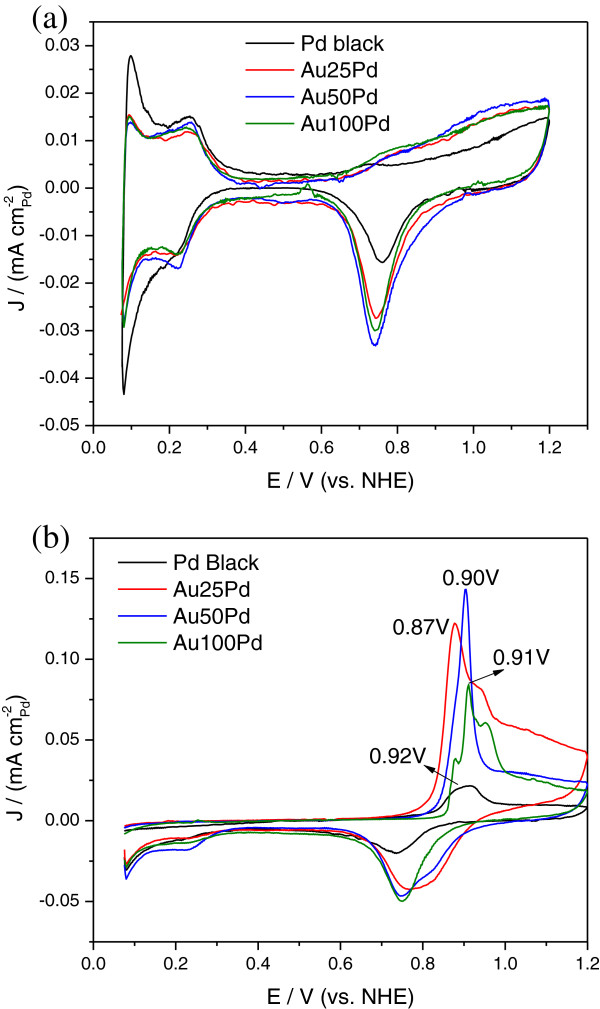
**Electrochemical properties of the Au/Pd and Pd black catalysts. **(**a**) CV curves and (**b**) CO-stripping CV curves of the Au/Pd and Pd black nanoparticles in 0.1 M HClO_4 _solution from 0.075 to 1.2 V. The currents are normalized to the ECSA of Pd.

Electrochemical properties of the Au/Pd and Pd black catalysts were evaluated in Figure 5. In the CV curves shown in Figure 5a, the current density (*J*) has been normalized to the electrochemical surface area (ECSA). ECSA (m^2^ g_Pd_^-1^) was calculated by integrating the hydrogen adsorption peak from the CV curves after correcting the double-layer charges [24]. In the anodic scan direction, the Au50Pd NPs show slightly higher Pd oxidation peak current than those of other catalysts even though the onset of Pd oxidation is postponed. Consequently, reduction of the PdO or PdOH formed during the anodic scan occurs at a slightly higher potential during the subsequent cathodic scan.

The above-observed results might be due to the electronic interaction between the Pd and Au and the geometric effect (or so-called ensemble effect [36]). For many surface reactions, a certain number of active sites are required. Ensemble of active sites on the catalyst surface impacts reaction selectivity and activity. The XPS results have already demonstrated that electronic interaction between the Pd and Au may not be significant to yield such different adsorption behavior of oxygen-containing species on the Au/Pd NPs. Therefore, we simply attribute the effect of different Au cores in the Au/Pd NPs to the geometric contribution.

This geometric effect is further confirmed and demonstrated by the CO-stripping results in Figure 5b. The CO coverages (Au25Pd = 0.88; Au50Pd = 0.94; Au100Pd = 0.9; Pd black = 0.78) calculated according to reference [37] are slightly different for different samples but close to unity. The Au25Pd displays the lowest CO oxidation potential at 0.87 V compared to the Pd black (0.92 V), Au50Pd (0.90 V), and Au100Pd (0.91 V). The availability of higher coordinated Pd sites (the most stable configuration) might be slightly reduced for smaller particle size due to the ensemble effect. Therefore, the adsorption strength of CO may be reduced as manifested by a negatively shifted peak potential for the Au25Pd.

The facile oxidation of CO on the core-shell NPs at lower potential will translate to an enhanced FAO kinetic since the FAO oxidation pathway involving CO or CO-like species results in lower activities of catalysts [38,39]. Figure 3a shows that the Au25Pd demonstrates the highest area-specific current density (5.5 mA cm^-2^) in the forward scan direction, while the Pd black only shows a peak current of 3.5 mA cm^-2^. Besides, the specific activity of Au25Pd at 0.3 V (the normal working potential in a DFAFC) is slightly higher (0.93 mA cm^-2^) than that of the Pd black (0.85 mA cm^-2^). In addition, Au25Pd shows the highest peak potential (approximately 0.9 V) in both the anodic and cathodic scans, indicating that significant oxygen-containing species (e.g., hydroxyl) only form at higher potential, and therefore, the Au/Pd catalysts could remain active over a wider potential window without being poisoned by hydroxyl groups. This is further demonstrated by the chronoamperometry tests in Figure 3b. The Au25Pd and Au50Pd show the highest area-specific current density (normalized to the ECSA of Pd) initially and are able to maintain their superior stability even after 1 h at *ca*. 0.144 mA cm^-2^, which is significantly higher than that of the Pd black (0.0099 mA cm^-2^).

Durability of the Au/Pd NPs was evaluated under the AST protocol with potentials applied between 0.6V (5 s) and 0.95 V (5 s) up to 14,000 cycles. Figure 3c shows that the Au25Pd preserves almost 90% of its initial ECSA in the first 7,000 cycles and 71% after 14,000 cycles. However, the ECSA loss for the Pd black is 35% in the first 7,000 cycles and 62% after 14,000 cycles. Not only the Au25Pd but also other Au/Pd catalysts demonstrate better electrochemical durability in the long-term AST. It is well known that dissolution of Pd in acidic electrolytes starts from the formation of PdO or PdOH. As Figure 5a shows, Au25Pd can depress the adsorption of oxygen-containing species within the potential window during the cycling tests; therefore, ensemble effect originated from the unique morphologies of the Au core in the Au25Pd may contribute to its superior durability.

## Conclusions

We have demonstrated that by decreasing concentration of the Au solution, the hollow Au cores in our unique Au/Pd core-shell NPs were formed with smaller crystalline grains and highly porous structures. Results indicated that these Au/Pd catalysts show superior catalytic activities as ideal catalysts for formic acid oxidation. Furthermore, these Au/Pd catalysts show excellent electrochemical stability, CO oxidation ability and long-term durability. Particularly, the Au25Pd NPs synthesized in this study present the best catalytic properties due to their unique structure. The hollow and porous gold cores tuned by reduced Au concentrations in the core-shell structures may influence Pd distribution and morphologies on the Au core. These remarkable properties make the Au/Pd NPs the promising catalysts for DFAFCs.

## Abbreviations

AST: Accelerated stress test; CV: Cyclic voltammetry; ECSA: Electrochemical surface area; FAO: Formic acid oxidation; NHE: Normal hydrogen electrode; NPs: Nanoparticles; TEM: Transmission electron microscopy; UV–vis: Ultraviolet–visible spectroscopy; XRD: X-ray diffraction; XPS: X-ray photoelectron spectroscopy.

## Competing interests

The authors declare that they have no competing interests.

## Authors' contributions

CHsu and FL designed and carried out the experiments and wrote the paper. CHuang and YH participated in the experiments and discussion. All authors read and approved the final manuscript.
